# AI-Guided Inflatable Neck Brace for Personalized Cervical Support

**DOI:** 10.3390/s26102928

**Published:** 2026-05-07

**Authors:** Abderrezaq Chemmami, Lyamine Guezouli, Aymen Ahmed Houasnia, Nabil Djenfi, Mohammed Amine Merzoug, Meriem Outtas, Djallel Eddine Boubiche, Homero Toral-Cruz, Rafael Martínez-Peláez, Francisco Méndez-Martínez, Manuel May-Alarcón

**Affiliations:** 1 LEREESI Laboratory, HNS-RE2SD, Batna 05000, Algeria; chemmami.abdrezak@hns-re2sd.dz (A.C.); houasnia.aymen.a@hns-re2sd.dz (A.A.H.); 2 Medicine Department, University of Batna 2, Constantine Road, Fesdis, Batna 05078, Algeria; n.djenfi@univ-batna2.dz; 3 Computer Science Department, University of Batna 2, Batna 05078, Algeria; amine.merzoug@univ-batna2.dz; 4 Univ. Rennes, INSA Rennes, CNRS, IETR-UMR 6164, 35700 Rennes, France; 5 Departamento de Ingeniería y Tecnología, Universidad Autónoma del Estado de Quintana Roo, Chetumal 77019, Mexico; 6 Unidad Académica de Computación, Universidad Politécnica de Sinaloa, Mazatlán 82199, Mexico; 7 Departamento de Ingeniería de Sistemas y Computación, Universidad Católica del Norte, Antofagasta 1270709, Chile; 8 Facultad de Ingeniería y Arquitectura, Universidad Autónoma del Carmen, Ciudad del Carmen, Campeche 24180, Mexicommay@pampano.unacar.mx (M.M.-A.)

**Keywords:** cervical disc herniation, AI-powered neck brace, personalized support, neck pain management, intelligent rehabilitation, wearable technology

## Abstract

Many people suffer from cervical disc herniation, which significantly affects the lives of individuals, causing chronic pain and functional limitations. This paper presents the development and evaluation of an AI-powered inflatable neck collar designed to provide personalized and adaptive support for individuals experiencing neck pain, particularly those with disc herniations. The system seamlessly integrates motion sensors, a robust AI model trained on a dataset of MRI scans, and a custom-designed inflatable collar. The AI model accurately detects disc herniations and segments vertebral structures, enabling real-time, targeted inflation adjustments based on the user’s unique anatomy, posture, and movements. A user-centered design approach ensures a seamless and intuitive user experience, allowing for personalized profile management, control over inflation levels, and data logging for tracking progress. Extensive simulations using 3D models and real-time data flow systems validated the effectiveness of the AI-guided system. Results demonstrated accurate detection and segmentation of disc herniations, robust real-time response, and adaptability to user needs. The proposed system, reviewed and validated by a neurosurgeon, demonstrates significant potential as a novel and effective solution for personalized treatment of neck pain, particularly in cases of disc herniation. Further development and research will focus on expanding the dataset to improve fairness and accuracy for diverse demographics and increasing the robustness and generalizability of the system.

## 1. Introduction

Cervical disc herniation is a prevalent condition that significantly affects the lives of many individuals, often leading to chronic pain and functional limitations. This condition occurs when the intervertebral discs in the cervical spine, which act as cushions between the vertebrae, become damaged or degenerated. The cervical spine comprises seven vertebrae, labelled C1 to C7, which connect the base of the skull to the upper back. Between each vertebra is an intervertebral disc, a cartilaginous cushion that serves as a shock absorber for the spine. These discs have two main parts: a soft, gel-like core called the nucleus pulposus, and a tough, fibrous outer ring called the annulus fibrosus. These discs allow a full range of movement, from bending and twisting to more complex motions. However, over time, the intervertebral discs in the cervical spine can lose water content and elasticity, a process known as disc degeneration. When the outer ring weakens due to age or repetitive stress, it may tear, allowing the nucleus pulposus to push out or herniate. Herniation can occur gradually as the disc degenerates, or it may be sudden due to a traumatic event such as a fall or whiplash injury. Once herniation occurs, the nucleus pulposus can press on surrounding spinal nerves or the spinal cord, leading to various neurological symptoms.

Diagnosing cervical disc herniation involves a thorough evaluation that includes a detailed medical history and physical examination. Physicians assess reflexes and muscle strength to identify any neurological deficits. Imaging studies, particularly magnetic resonance imaging (MRI), play a crucial role in confirming the diagnosis and determining the extent of nerve compression. MRI is especially valuable because it provides detailed images of soft tissues, allowing healthcare providers to visualize changes in disc structure and height. Treatment for cervical disc herniation typically begins with conservative management strategies. Most patients respond well to non-surgical approaches that may include activity modification, medications, physical therapy, and spinal injections. An important aspect of this conservative management is the use of neck braces, which provide essential support during the healing process. By stabilizing the cervical spine, neck braces help alleviate pain and limit movements that could exacerbate symptoms or lead to further injury. This stabilization is particularly beneficial in the early stages of treatment when inflammation and discomfort are most pronounced [1].

### 1.1. Research Motivation

The clinical effectiveness of rigid collars in managing conditions like cervical disc herniation has been questioned. Some studies suggest that soft collars may be equally effective in providing symptom relief without the drawbacks associated with rigid immobilization [2]:

- Excessive immobilization: Rigid collars are highly restrictive, significantly limiting neck movement [3].
- Musculoskeletal problems: Prolonged use can lead to muscle atrophy (weakening) and stiffness in the neck.
- Hindrance to daily activities (negative impact on quality of life): The high level of restriction can make it difficult for patients to perform routine tasks. Difficulty with tasks can lead to patient frustration and a decreased quality of life.
- Respiratory risks (restriction of breathing patterns): They pose risks of breathing difficulties and potential airway obstruction. Respiratory risks arise because the braces restrict normal breathing mechanics.
- Increased risk for vulnerable patients: The risk of breathing problems is particularly concerning for individuals who already have pre-existing respiratory conditions [4].

Despite their advantages in comfort over rigid alternatives, conventional soft collars possess their own notable limitations that motivate the need for an intelligent, adaptive system:

- Insufficient or non-targeted support: Soft collars offer minimal mechanical support, acting primarily as a kinesthetic reminder rather than providing targeted stabilization to specific, affected vertebral levels identified in medical imaging.
- Static and unresponsive: They provide a fixed level of cushioning. They cannot dynamically adjust based on the user’s real-time posture (e.g., sitting vs. standing), movement patterns, or changing therapeutic needs.
- Lack of personalization: Beyond basic sizing, they do not incorporate individual anatomical data, such as herniation severity from an MRI, to tailor the support provided.

These limitations highlight a clear need for a more personalized and adaptive system, which is where AI-driven analysis and control can offer significant advantages.

In their study, Fu et al. [5] introduced an automated AI system that quantifies the volume of cervical disc herniation from sagittal MRI images, achieving an average measurement error of only 5.8% compared to manual assessments. This approach reduces inter-observer variability and provides a standardized evaluation method to assist spine surgeons in diagnosis and treatment. While AI has been studied extensively in the context of spine surgery, with a particular emphasis on improving diagnostic accuracy, surgical planning, and intraoperative guidance [6,7,8], its use alongside non-surgical interventions, such as neck braces, remains comparatively under-explored.

To address this research gap, we propose the integration of AI within non-surgical procedures, specifically through the development of a neck brace that provides accurate and personalized cervical support. This approach has been developed with the support and medical advice of a neurosurgeon.

The integration of AI into the assessment and management of cervical disc herniation represents a significant advancement in improving clinical practices and patient care outcomes. Similar to recent AI-driven wearable systems that unobtrusively monitor user posture in real time to support musculoskeletal health [9], our system aims to extend such concepts toward active, adaptive support for cervical pathologies.

### 1.2. Research Methodology and Contribution

By combining the comfort and flexibility of soft neck collars with the precision and adaptability of AI technology, this work introduces an innovative approach to cervical support through the development of an AI-guided inflatable neck collar. Our proposed system utilizes patient-specific cervical MRI scans, which are conveniently uploaded via an accompanying mobile application. These medical images serve as the input for an integrated YOLOv8 deep learning model. This model is specifically trained to perform a detailed analysis, identifying the precise location and characteristics of disc herniations while also segmenting the relevant cervical vertebrae (C2–C7). Based on this objective anatomical assessment, the AI module formulates personalized recommendations for optimal inflation settings across different zones of the neck collar. This process ensures that the therapeutic support is directly tailored to the individual’s diagnosed condition.

The design also incorporates user-centric flexibility; individuals can use the mobile interface to manually fine-tune the inflation levels, allowing them to prioritize their subjective comfort alongside the AI’s guidance. To enhance user understanding and control, the application provides a real-time 3D visualization of the collar, offering immediate visual feedback on how adjustments affect its form.

This synergistic approach—merging (i) AI-driven analysis of medical data with user-controlled customization, (ii) input from integrated motion sensors for dynamic responsiveness, and (iii) clear visual feedback—aims to achieve both enhanced clinical efficacy and superior user comfort.

The key contributions of this paper can be summarized as follows:

- AI-driven personalization engine for inflatable cervical support: We introduce a system that integrates a YOLOv8 model, trained for multi-task MRI analysis (disc herniation detection and vertebral segmentation), to directly inform and personalize the inflation levels of a multi-zone inflatable neck collar.
- Real-time adaptive support through sensor fusion and AI: The system combines data from embedded motion sensors with the initial AI-derived anatomical insights to dynamically adjust collar inflation, offering responsive support that adapts to user posture and movement.
- User-centric mobile interface with integrated 3D visualization for enhanced therapeutic engagement: We designed a mobile application that empowers users by allowing MRI uploads, providing clear visual feedback of the AI’s analysis and collar adjustments via a real-time 3D model, and enabling manual control.
- Comprehensive system validation and performance assessment: The proposed system was rigorously evaluated, demonstrating strong performance from its core components. The AI model achieved a recall of 80% in detecting confirmed disc herniations and an exceptional mAP@0.5 of 0.995 for vertebral segmentation on our test dataset. Furthermore, the system’s adaptive control logic was validated through detailed 3D simulations, confirming its designed responsiveness to postural changes.

### 1.3. Structure of the Paper

The remainder of this paper is organized as follows. Section 2 reviews the existing literature on neck braces and relevant AI applications. Section 3 presents the proposed system design and architecture, covering its functional and non-functional requirements, AI-powered MRI analysis, hardware and software design, as well as the specific tools and methods used for implementation. Section 4 describes the experimental setup, presents the AI model’s performance results, and discusses the simulated system-level responses. Section 5 provides a discussion of the findings, compares them with existing approaches, and outlines the study’s limitations. Finally, Section 6 concludes the paper and highlights potential directions for future research.

It is important to note that this work should be interpreted as a computational proof-of-concept. The primary objective is to demonstrate the feasibility of the integrated AI-driven control logic through simulation and to establish a rigorous technical foundation for subsequent development. The system has not yet been validated on a wearable physical prototype, and the results presented herein are intended to motivate and guide the next stages of hardware fabrication, prototype testing, and eventual clinical evaluation.

## 2. Related Work

Traditional technology-driven neck braces incorporate engineering mechanisms for various healthcare purposes. For instance, powered neck braces, which are mainly characterized by the incorporation of motors, are used to assist in postural correction [10] and rehabilitation of patients with cervical disc degeneration [11], neurological disorders [12,13,14], and post-traumatic brain injury [15]. Another existing mechanism, parallel manipulator-based neck braces, are robotic structures composed of closed kinematic chains that allow precise motion control for posture control [16], rehabilitation [17,18], and even assistance with head–neck movements for daily activities [19].

Sensor-based neck brace technology, which incorporates sensors such as accelerometers, gyroscopes, and electromyography electrodes, is mainly used for measurement and real-time activity monitoring. Similarly, wearable sensing has been explored to monitor human states in real time, for example, classifying mental fatigue in construction equipment operators with a deep learning model using wearable EEG sensors [20,21,22,23]. Such works highlight the growing role of AI-enhanced wearable devices in supporting health and safety, which aligns with our proposed AI-guided inflatable neck brace for cervical support.

While these traditional technology-driven neck braces provide precise motion control and real-time monitoring, they generally lack the ability to learn and optimize treatments based on individual patient data over time [24,25].

To remedy this, AI has been used in recent advances in neck brace technologies to enhance active rehabilitation by enabling adaptive, data-driven adjustments tailored to the specific needs of each patient. For instance, Lozano et al. [26] present an active neck orthosis that employs a parallel mini-robotic device with a sliding mode control strategy and a computer vision interface for real-time range of motion monitoring to assist patients with cervical musculoskeletal disorders. Similarly, Chang et al. [27] introduce a bio-inspired robotic neck brace that utilizes a head-mounted deep learning-based eye-tracker technology from Pupil Labs to assist users with poor head control by mimicking natural head movements in response to a gaze direction. In their study, Puri et al. [28] and Arunkumar et al. [29] both focus on IoT-integrated neckbands equipped with accelerometers and gyroscopes to monitor neck positions, generating alerts for improper postures. They also employ machine learning to improve accuracy. Additionally, Carmona et al. [30] propose a novel wearable origami device designed to protect the head and cervical spine during falls, utilizing an embedded logistic regression algorithm and sensors to detect falls and deploy the protective structure rapidly.

Although the aforementioned AI-driven technologies [26,27,28,29,30] provide valuable support for posture correction, movement assistance, rehabilitation, or fall protection, they do not typically leverage deep analysis of patient-specific medical imaging like MRI to address pain originating from specific cervical vertebral pathologies such as disc herniation. To effectively bridge this gap, we propose a personalized AI-guided inflatable neck collar. Our system is distinct in its approach of utilizing MRI scan analysis to inform targeted, adjustable support directly aimed at alleviating pain and supporting the affected cervical structures.

## 3. System Design and Implementation

We begin with a general overview and architectural description of the system (Section 3.1), followed by a discussion of the key functional and non-functional requirements that guided its development (Section 3.2). We then detail the AI-powered MRI analysis module (Section 3.3) before addressing the hardware and software aspects of the system (in Section 3.4 and Section 3.5, respectively).

### 3.1. System Overview

**Figure 1:**
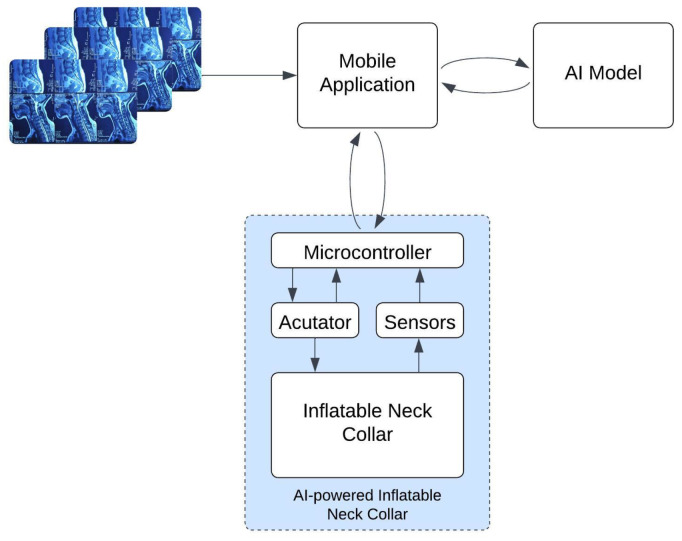
Architecture and workflow of the AI-guided inflatable neck brace. Sensors collect posture data, while the user can input scan images via the app. The AI model analyzes this data to recommend adjustments, and the ESP32 microcontroller controls the actuator to provide real-time, adaptive support.

The proposed system is designed to provide personalized and adaptive cervical support by leveraging the synergy between patient-specific anatomical data and real-time motion sensing. The operation is structured as a hierarchical data fusion framework, where high-level diagnostic insights from MRI scans guide the low-level, real-time actuation of the brace. As illustrated in the architecture and workflow diagram (Figure 1), the system integrates two primary data streams through the following implementation logic:

- AI-Derived Static Baseline Profile ($S_{\mathrm{init}}$): Upon uploading the cervical MRI scans, the AI engine identifies the presence and level of disc herniations. The system uses a spatial mapping table to assign an Initial Support Value ($S_{init}$) to specific inflatable zones. For instance, a detected herniation at C5–C6 prioritizes the lower posterior chambers. This value is scaled from zero to one, where one represents the maximum therapeutic pressure (2.0 PSI) and zero represents the baseline contact pressure (0.5 PSI).
- Sensor-Driven Dynamic Multiplier ($M_{\mathrm{dyn}}$): While the brace is worn, the IMU samples the neck’s orientation at 50 Hz. The control logic processes these data to calculate a Dynamic Multiplier ($M_{dyn}$) based on postural triggers. For example, if the system detects sustained neck flexion (angle $\theta >20^{\circ }$ for t>5 s), $M_{dyn}$ is increased proportionally to the degree of deviation to provide corrective stabilization.
- Hierarchical Fusion and Actuation: The final commanded pressure for each independent chamber ($P_{target}$) is calculated by synthesizing the diagnostic baseline with the real-time multiplier using the following support intensity formula:

(1) $$P_{target}=P_{min}+\left(P_{max}-P_{min}\right)\times \left[S_{init}\cdot M_{dyn}\right]$$

where:

- $P_{min}$ is the baseline contact pressure (0.5 PSI).
- $P_{max}$ is the maximum safety limit (2.0 PSI).
- $S_{init}$ is the diagnostic priority (0 to 1) derived from the MRI analysis.
- $M_{dyn}$ is the real-time postural adjustment factor.

This logic ensures that the brace remains anchored to the patient’s clinical diagnostic data while responding dynamically to their physical context. The overall architecture comprises several interconnected key components:

- Mobile application: Serves as the primary user interface for data input (MRI uploads) and manual override control.
- Embedded sensors (Sensing subsystem): The IMU array provides the closed-loop postural feedback necessary for $M_{dyn}$ calculation.
- AI engine: The YOLOv8 model serves as the “clinical brain,” interpreting medical imaging to personalize the brace’s response.
- Central control unit (Microcontroller): The ESP32 synthesizes the AI recommendations and sensor data to command the actuator subsystem.
- Actuator (Inflatable collar mechanism): A miniature air pump and manifold of valves translate the digital commands into physical inflation.

Critically, the proposed system is designed as a Clinical Decision Support System (CDSS), embodying a strict Human-in-the-Loop architecture. The AI engine does not autonomously initiate high-pressure therapy. Instead, it generates a “Proposed Inflation Profile” based on the MRI analysis, which the attending clinician must review and explicitly “Authorize” via the mobile application before any therapeutic actuation begins. This design ensures that the human expert remains the final authority and the responsible party for all therapeutic decisions, with the AI serving strictly in a supportive, advisory capacity.

### 3.2. Functional and Non-Functional Requirements

The design of the AI-guided inflatable neck brace was based on key functional and non-functional requirements to ensure efficacy, safety, and usability.

#### 3.2.1. Functional Requirements

- MRI scan ingestion: The system must allow users to upload cervical MRI scans via the mobile application.
- Disc herniation detection and vertebral segmentation: The AI model must accurately detect the presence and location of cervical disc herniations from the ingested MRI scans. The model must also accurately segment the cervical vertebrae (C2–C7) from the MRI scans to establish anatomical context.
- Generation of personalized inflation recommendation: Based on the AI analysis, the system must generate personalized inflation level recommendations for different zones of the inflatable neck collar (if multi-zone) or for the overall collar.
- Automated collar adjustment: The system must be able to automatically adjust the collar’s inflation to the AI-recommended levels via the controlled actuation mechanism.
- Manual user control: Users must be able to manually override AI recommendations and adjust collar inflation levels via the mobile application according to their comfort.
- Real-time posture and movement monitoring: The system must continuously monitor user neck posture and significant movements using embedded motion sensors.
- Dynamic adaptive support: Based on real-time sensor data and AI-derived insights, the system must dynamically adjust collar inflation to provide adaptive support during various user activities.
- 3D visualization: The mobile application must provide a real-time 3D visualization of the neck collar, reflecting its current inflation state and, ideally, its interaction with a simplified cervical spine model.
- User profile management: The system must allow users to create and manage personal profiles to store their settings and historical data.
- Data logging: The system must log relevant data, such as AI recommendations, user adjustments, collar usage duration, and potentially posture/movement patterns, for progress tracking and potential future analysis.

#### 3.2.2. Non-Functional Requirements

- Comfort: The inflatable collar must be designed to be comfortable for extended periods, minimizing skin irritation and pressure points.
- AI model accuracy: The AI model must achieve high accuracy in detecting cervical disc herniation and cervical vertebrae segmentation.
- Real-time response: For dynamic adaptive support, the system must respond to significant changes in user posture/movement by adjusting collar inflation within seconds.
- Safety: The internal pressure of the inflatable chambers is strictly regulated within a safe operating range of 0.5 to 2.0 PSI. This range was defined based on a combination of clinical neurosurgical advice and biomechanical safety thresholds. Specifically, the lower limit of 0.5 PSI (~25.8 mmHg) ensures that the collar maintains structural contact and a kinesthetic reminder for the user without causing skin ischemia, as it stays near the mean capillary refill pressure. The upper limit of 2.0 PSI (~103.4 mmHg) is designed to provide significant mechanical stabilization for the C2–C7 vertebrae while remaining safely below the average arterial pressure. This prevents excessive pressure on the carotid triangle and avoids potential compression of the jugular veins or the airway, ensuring comfort and safety during prolonged wear.
- Safety: The collar inflation mechanism must operate within predefined safe pressure limits to prevent overinflation and ensure user safety. These limits should be determined in consultation with medical professionals. Additionally, materials used in the inflatable collar that come into contact with the user’s skin should be biocompatible and non-irritating.
- Reliability: Hardware (sensors, microcontroller, actuator, and collar) and software components must operate reliably. The collar must also provide sufficient battery life for at least one day of typical use on a single charge.
- Usability: The mobile application interface must be intuitive, accessible, and easy to navigate for the demographic of the target user, including individuals with potential physical limitations or varying technological knowledge.
- Data privacy and security: All patient-identifiable information and medical data (MRI scans, usage logs) must be stored and transmitted securely, in accordance with relevant data protection regulations such as GDPR (HTTPS, database encryption, secure authentication, etc.).

### 3.3. MRI Scan Analysis

This section outlines the imaging data workflow, from acquisition and preprocessing to analysis using the AI model.

#### 3.3.1. Dataset Acquisition and Preparation

A retrospective dataset comprised of cervical MRI scans was sourced from the University Hospital of Batna, Algeria. Ethical considerations were paramount; the data was collected retrospectively and fully anonymized according to hospital regulations. The dataset includes 120 unique patients (totaling 250 high-quality sagittal T2-weighted slices). This diversity ensures the AI model generalizes across varied stages of spinal degeneration and differing neck anatomies. To ensure clinical relevance and suitability for AI model training, the collected dataset was meticulously reviewed by a collaborating neurosurgeon (co-author Dr. Nabil Djenfi). All scans were acquired using a Siemens Magnetom Symphony 1.5T MRI system with standardized imaging parameters: Repetition Time (TR) = 3500 ms, Echo Time (TE) = 100 ms, and a slice thickness of 3.0 mm. Images were selected based on stringent criteria, prioritizing high-quality scans with clear visibility of any disc herniation and the surrounding vertebral structures. The dataset was curated to include cases both with and without disc herniation to facilitate balanced training for the detection task. The final dataset has a class distribution of 1/3, and ground-truth labeling was performed by Dr. Djenfi to ensure the highest diagnostic accuracy for the training labels.

A retrospective dataset comprised of cervical MRI scans was sourced from the University Hospital of Batna, Algeria. Ethical considerations were paramount; the data was collected retrospectively and fully anonymized according to hospital regulations before any handling or storage, ensuring patient privacy. The project’s data management, including secure storage and access control, was considered by implementing robust security measures such as HTTPS encryption and granular access rules.

To ensure clinical relevance and suitability for AI model training, the collected dataset was meticulously reviewed by a collaborating neurosurgeon (co-author Dr. Nabil Djenfi). Images were selected based on stringent criteria, prioritizing high-quality scans with clear visibility of any disc herniation and the surrounding vertebral structures. To enhance the focus on the region of interest and reduce computational overhead, the selected images were cropped to isolate the cervical spine (vertebrae C2–C7). The dataset was curated to include cases both with and without disc herniation to facilitate balanced training for the detection task. The final dataset has a class distribution of 1/3.

The comprehensive annotation of the MRI dataset was performed using Makesense.ai (Makesense.ai: https://www.makesense.ai/ (accessed on 7 April 2026)). This platform facilitated the precise delineation of disc herniations using rectangular bounding boxes and the segmentation of cervical vertebrae with polygonal annotations.

- Disc herniations: Annotated with rectangular bounding boxes (Rect Annotator) in YOLO format (*<class-index><center-x><center-y><width><height>*).
- Cervical vertebrae (C2–C7): Segmented using polygonal annotations (Polygon Annotator) and exported in COCO JSON format.
- Vertebral centers: Marked using the Point Annotator and exported as a CSV file (center-label, cx, cy, image-name, width, height) to aid in spatial localization.

**Figure 2:**
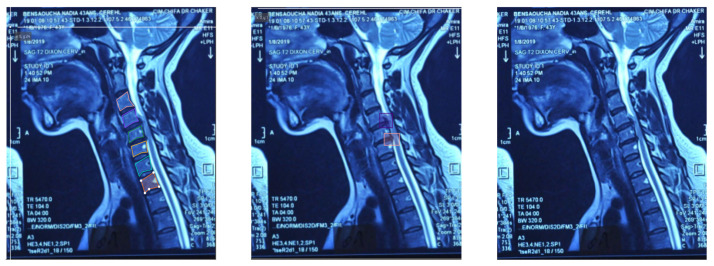
Labelled and raw data exemples.

**Figure 3:**
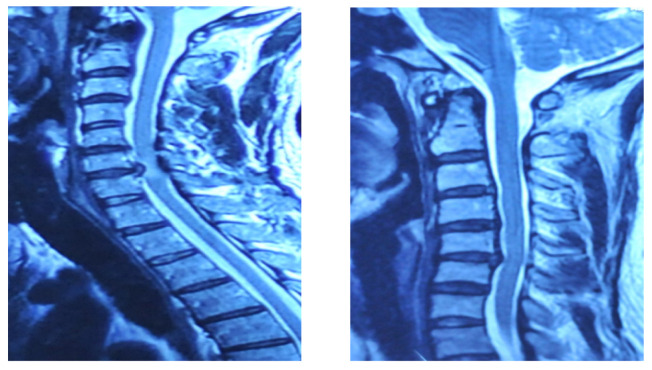
Examples of cropped MRI images.

This detailed annotation, which took approximately one month, underwent multiple review cycles with the neurosurgeon to ensure medical accuracy and consistency. Figure 2 illustrates an example of a raw MRI image utilized in this study, while Figure 3 shows examples of cropped images.

#### 3.3.2. Data Preprocessing and Augmentation

Following selection and annotation, the cropped MRI images underwent a preprocessing pipeline to optimize them for compatibility with the YOLOv8 model and to enhance training efficiency. This pipeline included:

- Resizing: Images were resized to standardized dimensions of 640 × 640 pixels to ensure consistent input for the AI model.
- Normalization: Pixel values were scaled to the [0, 1] range by dividing by 255. This normalization step is standard in YOLOv8’s preprocessing pipeline and aids in training stability and convergence.

The dataset was then split into training and validation sets. Of the data, 75% was allocated for training the YOLOv8 model, and 25% for validation during training to monitor performance and prevent overfitting. To evaluate the trained model’s generalization capability, a separate test set consisting of external MRI scan images—depicting cervical spine conditions and downloaded from public web sources—was used. This test set ensured the model was evaluated on entirely unseen data, providing a realistic assessment of its robustness against variations not present in the training dataset.

To improve the model’s ability to generalize to variations in MRI acquisition conditions, patient positioning, or anatomical anomalies, data augmentation techniques were applied during model training to the training dataset. These techniques included:

- Random rotation: Images were randomly rotated within a range of +/−5 degrees.
- Scaling: Images were randomly scaled along both X and Y axes within a range of +/−2%.

These augmentations aimed to create a more diverse training set, thereby enhancing the network’s robustness in detecting herniation instances across a wider range of imaging scenarios.

In addition to these training-time augmentations, a mirrored-image processing technique was utilized during the evaluation phase to further enhance robustness against potential left-right asymmetries in MRI scans (the results of this technique are detailed in Section 4.5.3).

#### 3.3.3. AI Engine

The AI engine employs a customized YOLOv8 model, a state-of-the-art object detection and segmentation algorithm known for its optimal balance of speed and accuracy. The YOLOv8 architecture generally consists of:

- Backbone (e.g., CSPDarknet): For extracting rich feature hierarchies from the input images.
- Neck (e.g., PANet or BiFPN): For aggregating features from different backbone stages to enhance multi-scale detection.
- Head: Responsible for the final outputs, including rectangular bounding boxes for disc herniation detection, class probabilities, and polygonal segmentation masks for vertebral structures.

YOLOv8 was specifically selected over alternative architectures such as Mask R-CNN and Faster R-CNN for several key reasons. First, YOLOv8 employs a single-stage architecture that performs object detection (bounding boxes) and instance segmentation (polygon masks) simultaneously in a single forward pass through the network. In contrast, two-stage detectors like Mask R-CNN require a separate region proposal network (RPN) pass followed by a refinement stage, making them significantly more computationally expensive and slower. This speed advantage is critical for the intended real-time integration with the embedded ESP32 microcontroller hardware. Second, despite its speed, YOLOv8 maintains superior accuracy for detecting small anatomical features such as disc protrusions, which are subtle and spatially small targets within MRI scans. Third, YOLOv8’s unified detection and segmentation pipeline is particularly well-suited for simultaneously localizing herniation regions and delineating the precise boundaries of cervical vertebrae (C2–C7), a dual-task that would require separate model pipelines in two-stage approaches. During the development phase, the model’s performance was optimized through a systematic hyperparameter tuning process using a grid search approach. We explored a range of critical parameters to ensure the highest diagnostic reliability:

- Learning Rates: Explored from 0.01 to 0.0001.
- Batch Sizes: Evaluated at 8, 16, and 32.
- Optimizers: Compared the performance of Stochastic Gradient Descent (SGD) and AdamW.
- Training Duration: Set to 300 epochs with an early-stopping patience of 50 epochs to prevent overfitting.

The criterion used to select the best configuration was the highest mean Average Precision (mAP@0.5) achieved on the validation set, as this metric effectively captures the model’s ability to balance localization accuracy and classification precision. The final optimized configuration utilized a learning rate of 0.01, a batch size of 16, and the AdamW optimizer.

The training was implemented using the PyTorch 2.6.0 framework (version 2.0.1) on an Nvidia GPU with CUDA 11 for accelerated computation (Nvidia, Santa Clara, CA, USA). The model’s weights were adjusted iteratively until it reliably converged, demonstrating consistent performance on the validation set. The trained YOLOv8 model generates detailed outputs for each analyzed MRI scan, including:

- Disc herniations: Bounding box coordinates, a confidence score for the detection, and the class label.
- Vertebrae (C2–C7): Segmentation masks delineating the precise boundaries of each vertebra.
- Derived information: From these primary outputs, the system further derives size, position, and directional information (e.g., vectors indicating herniation protrusion relative to vertebral bodies).

These outputs enable the system to accurately determine the location and extent of pathology and to focus the inflatable collar’s support precisely. For instance, the system can identify which intervertebral disc spaces are affected and the relative positions of the vertebrae, which directly informs the personalized inflation strategy.

### 3.4. Hardware Design

The hardware components of the system play a critical role in translating AI-driven recommendations and sensor feedback into physical support. These components include (i) the collar structure, (ii) the sensing array, and (iii) the control and actuation mechanisms.

#### 3.4.1. Inflatable Collar

**Figure 4:**
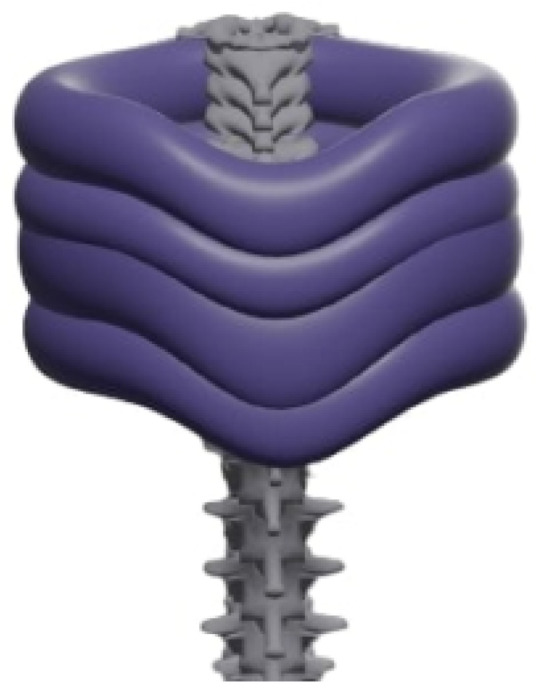
Inflatable mechanism of the neck brace.

The mechanical design of the inflatable neck collar prioritizes user comfort, targeted support, and reliable operation. Key design considerations include:

- Ergonomic support chamber: The core of the device is an inflatable chamber meticulously shaped to conform to the natural curvature of the cervical spine. This ergonomic design aims to provide a snug yet comfortable fit, distributing support effectively around the neck.
- Personalized fit: The chamber’s overall volume and pressure are adjustable, allowing the collar to be personalized for a wide range of neck sizes and individual therapeutic requirements (Figure 4).
- Inflation control: To facilitate precise and responsive adjustments, a reliable and easy-to-operate valve is integrated into the collar. This mechanism is designed to (i) prevent air backflow, maintaining the desired inflation level, and (ii) allow for fine-grained control over both the inflation and deflation processes. These valves are activated by actuators (detailed in Section 3.4.3) connected to the ESP32 microcontroller, enabling the AI engine and user inputs to modulate the collar’s support profile.
- Material Specifications and Durability: For both the 3D simulation and future physical prototyping, the collar is modeled as being constructed from medical-grade Thermoplastic Polyurethane (TPU). TPU was selected as the primary material due to its exceptional airtightness, which is essential for maintaining consistent pressure in the inflatable chambers without leakage. In terms of durability, TPU provides high tensile strength and abrasion resistance, ensuring the collar can withstand repeated inflation-deflation cycles and the mechanical stresses of daily use. Regarding skin compatibility and comfort, medical-grade TPU is biocompatible, hypoallergenic, and non-irritating, making it suitable for prolonged contact with the skin. Its flexibility allows the collar to remain soft and adaptable to the user’s movements while providing the necessary structural reinforcement.
- Power source: The wearable components of the collar are powered by a rechargeable battery. The selection criteria for the battery emphasize sufficient battery life for a full day of typical use, lightweight design to minimize user burden, and efficient charging capabilities to reduce downtime.

**Figure 5:**
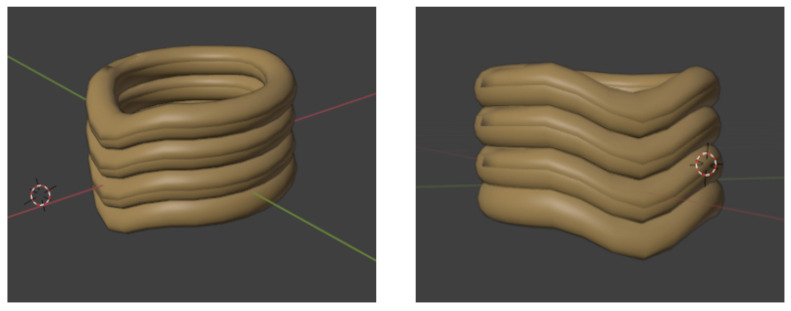
Inflatable collar design concepts.

A 3D representation of the inflatable collar design, illustrating its general form, is shown in Figure 5.

#### 3.4.2. Sensing Subsystem

The primary role of the sensing subsystem is to monitor the user’s neck posture and significant movements, providing dynamic data that can inform adjustments to the collar’s inflation profile, complementing the AI recommendations.

- Core motion sensing unit: The central component of this subsystem is an IMU (Inertial Measurement Unit). In particular, a single IMU (e.g., a commonly available unit like the MPU-6050 or similar, integrating a 3-axis accelerometer and a 3-axis gyroscope) would be strategically embedded within the collar structure, ideally at the posterior midline to best capture the overall motion and orientation of the cervical region.
  ‒ The accelerometer measures linear accelerations, including the static gravity vector, which is fundamental for determining orientation when the system is relatively still. It also detects dynamic accelerations indicative of user movements or external perturbations.
  ‒ The gyroscope measures the angular velocity (rate of rotation) around three orthogonal axes, providing information about how quickly the neck is turning, tilting, or flexing/extending.
- On-board data processing (for orientation estimation): Raw data streams (acceleration and angular velocity) from the IMU are processed by the ESP32 microcontroller (detailed in Section 3.4.3). To derive a stable and accurate estimation of the neck’s orientation (e.g., pitch for flexion/extension, roll for lateral bending), a sensor fusion algorithm is employed. Common lightweight algorithms suitable for embedded systems include Complementary Filters or Madgwick Filters, which combine accelerometer and gyroscope data to mitigate individual sensor drift and noise. The output would typically be Euler angles or quaternions representing the neck’s orientation relative to a calibrated neutral or resting posture.
- Input to adaptive control logic: The calculated orientation and processed motion data serve as real-time inputs to the adaptive control algorithms running on the ESP32. This enables dynamic adjustments to collar inflation based on predefined rules and thresholds. For instance:
  ‒ Sustained postural deviations: If the system detects prolonged neck flexion (e.g., pitch angle exceeding approximately 20–25 degrees) for a specified duration (e.g., 10–15 s), it could trigger a gradual increase in pressure to the anterior support elements of the collar, gently encouraging a return to a more neutral posture.
  ‒ Movement-specific support: For certain detected movements or activities (e.g., rapid head turns indicating a need for temporary increased stabilization), the system could momentarily adjust inflation to provide enhanced support.
  ‒ Comfort in neutral posture: Conversely, during periods of sustained neutral posture with minimal movement, the system must be designed to slightly reduce inflation levels for increased comfort while maintaining the baseline therapeutic support.
  The specific thresholds, response magnitudes, and timing for these adaptive adjustments are critical parameters that must be determined and refined through iterative user testing and clinical evaluation (as discussed in Section 5).
- Potential for integrated pressure sensing: For enhanced precision in delivering and maintaining the targeted inflation levels, the integration of miniature pressure sensor(s) within the inflatable chamber(s) is a key consideration for future prototypes. Such sensors would provide direct feedback to the ESP32, enabling a more robust closed-loop control system for the actuation mechanism, ensuring that the actual pressure accurately matches the desired pressure, whether derived from AI recommendations or real-time adaptive logic.

#### 3.4.3. Control and Actuation Subsystem

The central processing and control for the wearable hardware components are managed by an ESP32 microcontroller, selected for its robust processing capabilities, low power consumption, and integrated wireless connectivity (Bluetooth and Wi-Fi). The control logic embedded in the ESP32 is responsible for:

- Receiving target inflation parameters: Derived from the AI model’s analysis of the user’s MRI (Section 3.3) and manual adjustments made via the mobile application.
- Processing real-time sensor data: Utilizing data from the embedded motion sensors (Section 3.4.2) to inform dynamic adjustments based on the user’s posture.
- Orchestrating actuation: Commanding the miniature air pump and electronically controlled valves to achieve and maintain the desired inflation levels across the different collar zones.
- Communication management: Handling data exchange with the mobile application to send status updates and receive new user commands.

In the current architecture, the system operates as a closed-loop postural feedback system, where the IMU data (Euler angles and quaternions) serves as the primary feedback variable to adjust the stabilization profile in real-time. However, regarding inflation precision, the system currently employs a calibrated open-loop pressure control mechanism. In the absence of integrated pressure sensors, inflation accuracy is assessed by the control logic using a precisely timed actuation model. By knowing the air pump’s volumetric flow rate and the chamber volume, the ESP32 calculates the duration of pump activity required to reach the target pressure (0.5–2.0 PSI). While this time-based calibration was validated as effective within the simulation environment, future hardware iterations will integrate miniature MEMS pressure sensors within the inflatable chambers. This addition will enable a secondary, true closed-loop pressure control architecture, allowing the system to monitor and compensate for minor air leaks or external compression in real-time.

#### 3.4.4. Safety and Compliance

The system incorporates three complementary layers of safety to ensure patient protection and regulatory compliance. At the hardware level, mechanical overpressure relief valves are set at a maximum threshold of 2.2 PSI, providing a passive failsafe against any uncontrolled pressure buildup. At the software level, hard-coded PWM duty cycle limits are enforced within the ESP32 firmware, preventing the pump from exceeding safe actuation parameters regardless of software commands. At the user interface level, a dedicated “Emergency Deflate” button is prominently integrated into the mobile application, allowing the patient or caregiver to instantly and fully deflate all collar chambers. The overall system design was guided by the IEC 60601-1 (Medical Electrical Equipment Safety) and IEC 62304 (Medical Device Software Lifecycle Processes) standards to ensure a safe and structured development process.

### 3.5. Software Design

The software components of the system include a mobile application for real-time user interaction (Section 3.5.1) and a 3D simulation environment used for design validation and testing (Section 3.5.2).

#### 3.5.1. Mobile Application

The mobile application serves as the primary interface for patient interaction, data input, and system control. Its design philosophy prioritized intuitiveness, accessibility, and a positive user experience for a diverse target audience (including individuals with varying levels of technological familiarity and those experiencing neck pain). The core functionalities of the mobile application include:

- MRI scan management: Facilitates the secure upload of the user’s cervical MRI scans, which form the basis for the AI engine’s analysis (as detailed in Section 3.3).
- Personalized inflation control: Displays AI recommendations for collar inflation and allows users to precisely adjust these levels to achieve optimal therapeutic support and subjective comfort. The manual override capability is a crucial aspect of the user-centric design, empowering users to tailor the treatment to their immediate needs.
- Real-time monitoring and visualization: Provides real-time visual feedback on the collar’s status, including current inflation levels and battery status. A key feature is the integration of a 3D visualization of the neck collar (interacting with a simplified cervical spine model), enabling users to intuitively understand the impact of their adjustments (as illustrated in Figure 7).
- User profile and data management: Allows users to create and manage personalized profiles, storing individual settings, pressure preferences, and historical usage data. This data logging feature is designed to help users and potentially clinicians track treatment progress, pain patterns, and overall collar usage trends.
- User feedback and support channels: Incorporates mechanisms for users to provide feedback on their experience and access support information.

The application was developed using Flutter (Flutter: https://flutter.dev/ (accessed on 7 April 2026)), selected for its key advantages in cross-platform development. User data management, including profile information and usage logs, was handled via Firebase (Firebase: https://firebase.google.com/ (accessed on 7 April 2026)) as a backend solution. The latter offers robust features for data storage, authentication, and security, aligning with the non-functional requirements for data privacy and security.

#### 3.5.2. 3D Simulation Environment

The implementation of the system hardware (encompassing the inflatable collar structure, sensing subsystem, and ESP32-based control and actuation mechanisms) was addressed through a 3D simulation environment at this stage of the research. In particular, the aim was to:

- Test the logic of the AI-recommended inflation adjustments by observing their application on the virtual collar and its interaction with the simulated cervical spine.
- Evaluate the designed responsiveness of the collar’s inflation/deflation mechanisms to control signals.
- Provide a visual platform for understanding the potential impact of different support strategies, which also informed the design of the 3D visualization feature within the mobile application.

This simulation-based approach offered several advantages during the design and development phases:

- Cost-effective iteration: Significantly reduced development costs by minimizing the need for multiple expensive physical prototypes during early-stage design exploration.
- Rapid parameter testing: Enabled rapid testing and iteration of various design parameters (e.g., inflation patterns, chamber responses) and scenarios (e.g., different neck movements, simulated anatomical variations) without requiring physical modifications to a hardware prototype.
- Insight generation: The simulation facilitated the exploration of a wide range of neck movements, inflation distributions, and support scenarios, potentially revealing insights into collar performance and biomechanical interactions that might not be immediately evident through limited physical testing alone.

**Figure 6:**
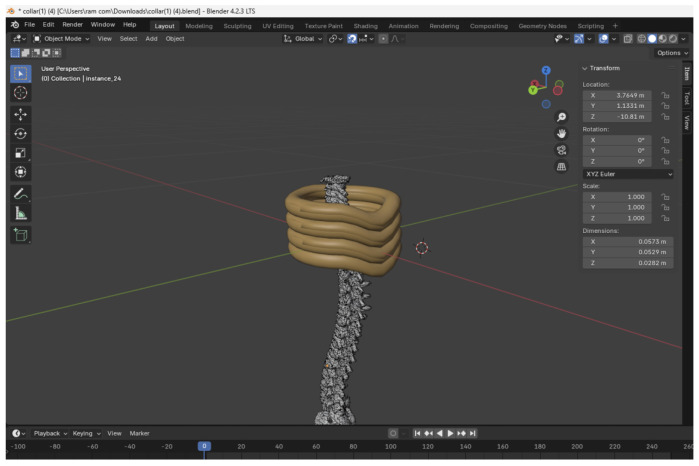
Cervical spine and neck brace simulation in Blender.

A detailed 3D model of the cervical spine and the inflatable neck collar served as the foundation for this simulated environment. This model, meticulously crafted to capture essential anatomical details and collar structural properties, allowed for the exploration of various inflation scenarios and their effects on simulated neck kinematics (see Figure 6).

While initial 3D modeling and animation sequences were developed using Blender (Blender: https://www.blender.org/ (accessed on 7 April 2026)), Three.js (Three.js: https://threejs.org/ (accessed on 7 April 2026)), an open-source JavaScript (version 0.160.0) library, was chosen for creating the interactive real-time simulation environment.

- 3D modeling and animation (Blender): The detailed 3D geometric models of the cervical spine and the inflatable neck collar were created and refined using Blender, an open-source 3D creation suite. Blender’s comprehensive modeling tools were leveraged to achieve visually accurate and adaptable representations, capturing the anatomy of the neck and the structure of the collar (Figure 6). Furthermore, Blender’s Python 3.14 scripting API was utilized for automating animation sequences, such as the collar’s inflation and deflation mechanisms and simulated neck movements. Custom scripts were developed to control animation parameters, enabling fine-tuned motion for realistic simulation scenarios.
- Interactive simulation environment (Three.js): To create an interactive, real-time simulation environment, particularly for testing dynamic responses and for potential integration with a web-based or mobile application-like interface, Three.js was employed. Models created in Blender were exported and then imported into the Three.js environment. This JavaScript library facilitated the rendering of the 3D scene and allowed for the dynamic manipulation of the collar model based on simulated AI commands or user inputs, replicating aspects of the system’s intended real-world interaction. This setup was crucial for analyzing the real-time interplay between the control logic and the 3D model’s behavior.

The rationale for employing Three.js, particularly for aspects interfacing with or emulating the mobile application’s visualization capabilities, was its:

- Web and mobile integration: Robust capabilities for rendering interactive 3D content directly within web browsers and facilitating easier integration or emulation of 3D views within mobile application frameworks. This was critical for testing real-time interaction between a simulated user interface (akin to the mobile app) and the 3D model’s behavior.
- Dynamic interaction: Suitability for creating dynamic, scriptable scenarios where parameters could be changed in real-time to observe the collar’s response, which is less straightforward to achieve interactively with Blender’s offline rendering focus alone.
- Seamless model conversion: Good compatibility with Blender, allowing for relatively seamless conversion and use of Blender-created models within the Three.js environment while retaining essential geometry and material properties for dynamic simulation.

This approach allowed for a critical pre-physical-prototype validation of the system’s core control logic and user interface concepts related to 3D visualization. The specific parameters governing the simulation setup, including the environment’s dimensions, lighting conditions, and camera configurations, are summarized in Table 1. The simulation also incorporated patient-specific anatomical data derived from MRI scan analysis to enhance the relevance and precision of the simulated scenarios.

Beyond its role as a simulation platform, the 3D digital twin of the C2–C7 cervical spine serves as the primary Explainable AI (XAI) interface of the system. Rather than presenting the clinician with opaque numerical recommendations, the digital twin provides a visual explanation of the AI’s diagnostic reasoning. Specifically, the XAI interface operates on the following parameters and customization logic:

- Pathology Highlighting: The specific vertebral segments (C2–C7) where the AI model detected disc herniations are visually highlighted on the 3D spine model (e.g., using distinct color coding), directly communicating the anatomical basis for the inflation recommendation.
- Calculated Support Vector Display: The magnitude and direction of the “Calculated Support Vectors” representing the required corrective pressure forces for each collar chamber are rendered as directional indicators on the 3D model, illustrating why a specific inflation profile was recommended.
- Patient-Specific Customization: The digital twin geometry and the displayed support vectors are adapted based on the patient-specific MRI analysis, ensuring the visualization reflects individual anatomy rather than a generic model.

This approach allows the clinician to verify the AI’s logic intuitively before authorizing the therapy, transforming the system from a “black box” into a transparent, human-interpretable decision support tool.

## 4. Performance Evaluation

The experimental setup was evaluated within a high-fidelity simulated environment designed to mimic real-world conditions. The evaluation focused on testing the trained YOLOv8 model for accurate detection and segmentation, as well as testing the AI-guided inflatable neck collar within this simulated environment.

### 4.1. System Effectiveness and Real-Time Responsiveness

A comprehensive evaluation process was conducted to assess the real-time response and effectiveness of the AI-guided adjustments within the inflatable neck collar. This evaluation involved a multi-pronged approach, utilizing (i) a combination of 3D simulation and (ii) a real-time data flow system designed to mimic real-world scenarios.

The real-time data flow system was created to replicate the collar’s functionality in a realistic setting. This system simulated:

- MRI scan data, introducing varied levels of quality and clarity to mimic real-world conditions.
- The real-time processing of this simulated data by the AI model, which generated recommendations for inflation levels based on detected disc herniations and vertebral segments.
- The transmission of these AI-generated recommendations to the virtual collar, leading to simulated adjustments in inflation levels.

The 3D simulation environment, which was constructed using Three.js, enabled virtual testing of the AI-guided adjustments. In particular, this simulation facilitated:

- Visualization of the collar’s behavior, demonstrating its inflation and deflation patterns, and their impact on the vertebral structures.
- Processing of simulated MRI scan data, mirroring real-world scenarios and their varying quality, and analyzing this data using the trained YOLOv8 model.
- Generation of real-time AI recommendations for inflation levels based on the detected disc herniations and vertebral segments.
- Simulating the application of these recommendations to the virtual collar, verifying that it inflates and deflates accurately in response to the AI’s instructions.

### 4.2. Accuracy and Safety of Inflation Mechanism

The real-time responsiveness and adaptive capabilities conceptualized for the proposed system were evaluated through a series of simulations, which aimed to assess the system’s ability to accurately implement inflation commands and the control logic’s capacity to dynamically adjust support based on simulated posture changes.

Simulations were also conducted to verify the accuracy, speed, and safety with which the designed inflation mechanism (as controlled by the simulated ESP32 and actuator logic) could respond to commands within the virtual environment.

- Achieving target pressure in simulation: When a target inflation pressure was commanded (either from a simulated AI recommendation or a mock manual input), the simulated collar system consistently achieved the target pressure within an average of *3 s*, with a simulated steady-state error of less than *5%*.
- Adherence to safety limit in simulation: Across all simulated inflation scenarios, the simulated controlled inflation mechanism successfully maintained pressures within the predefined safe operational limits of 0.5 PSI (pounds per square inch) to 2.0 PSI (as established in non-functional requirements).

### 4.3. Adaptation of Control Logic to Postural Changes

The real-time adaptive support of the system, specifically the responsiveness of its control algorithms to postural cues, was evaluated by simulating common postural changes and observing the intended collar inflation adjustments based on mock IMU data inputs. These simulations aimed to validate the control logic that would govern the physical device.

- Scenario 1: Simulated response of control logic to sustained neck flexion.
  ‒ Simulation setup: A scenario representing sustained neck flexion was created by providing a mock IMU pitch angle input to the simulated control system. This input changed from a neutral 0 degrees to 25 degrees over 3 s and was then maintained.
  ‒ System response: Upon detection of this sustained flexion input by the control algorithm (after a predefined threshold of *10 s*), the simulation demonstrated that the control logic would command an increase in the overall collar inflation pressure. The calculated target pressure increased from an initial 1.0 PSI to a supportive level of 1.5 PSI. The simulation indicated that the command for this corrective inflation adjustment would be generated by the algorithm within *0.5–1 s* from the initiation of the response.

- Scenario 2: Simulated response of control logic to change to resting position.
  ‒ Simulation setup: A transition to a resting posture was simulated by providing mock IMU pitch and roll angle inputs (near-zero values, minimal dynamic movement) to the simulated control system for *30 s*.
  ‒ System response: The simulation showed that after processing this input from the resting state, the control algorithm would command an adjustment of the simulated collar to a comfort profile. The calculated target pressure was reduced from a typical active support level of *1.2 PSI*. The simulation indicated this command for lower pressure would be generated by the algorithm approximately *1.0 s* after the resting state input was established.

### 4.4. User Interface and Interaction Evaluation

An initial evaluation of the mobile application’s UI and interaction experience was conducted through testing among 10 members of the development and research team.

**Figure 7:**
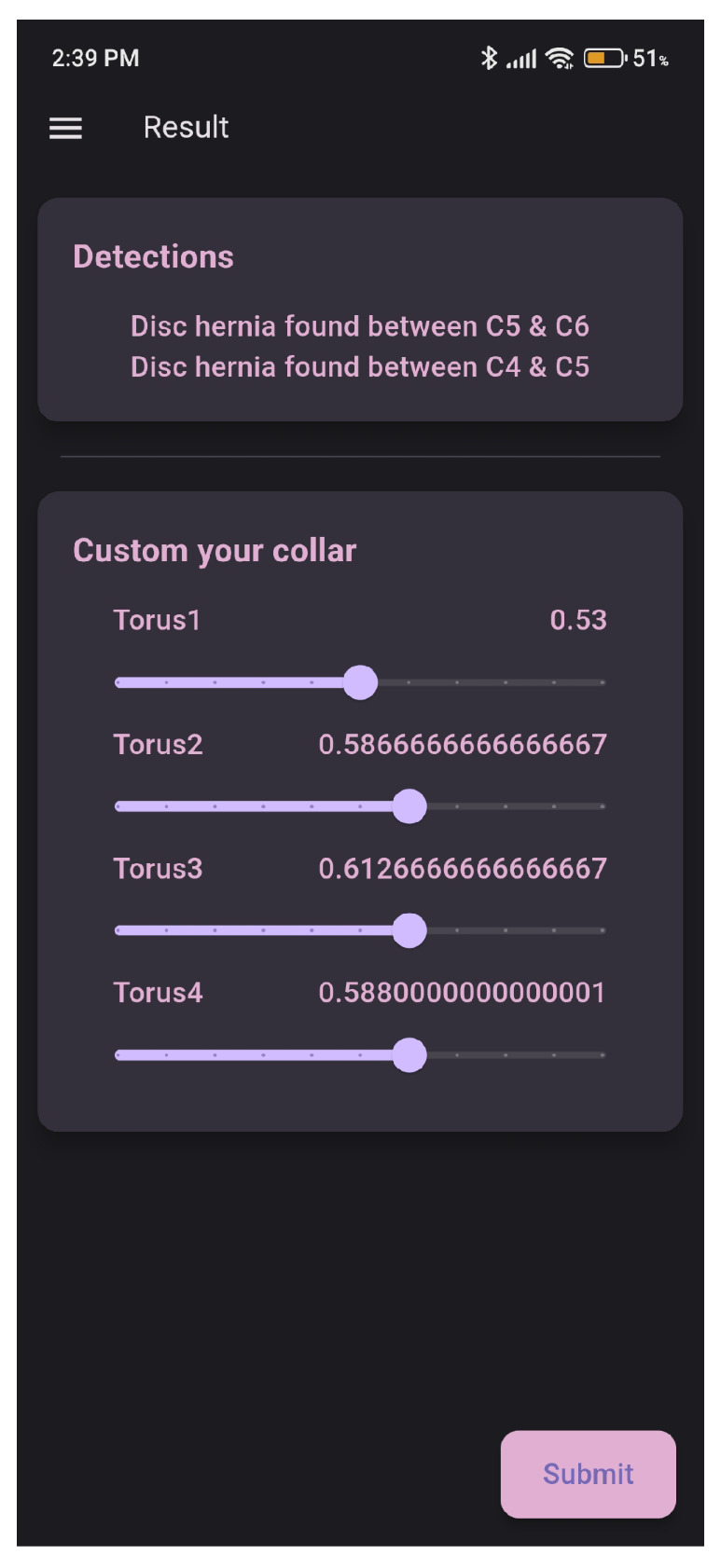
Mobile application interface showing results and 3D visualization.

- Ease of core task completion: Team members successfully navigated core tasks, including simulating MRI uploads, viewing mock AI recommendations, and manually adjusting simulated collar inflation via the app, with minimal instruction.
- Feedback on 3D visualization: The integrated 3D visualization of the neck collar (Figure 7) was reported as an attractive and intuitive feature, aiding in understanding collar adjustments.
- Overall usability: The general feedback indicated the application’s interface was clear and core functionalities were straightforward for the internal team, with no significant usability roadblocks identified in this phase.

### 4.5. AI Engine Evaluation

This section presents the performance evaluation of the AI engine in its core tasks of disc herniation detection and cervical vertebrae segmentation, as well as the evaluation of the mirrored image processing technique.

**Figure 8:**
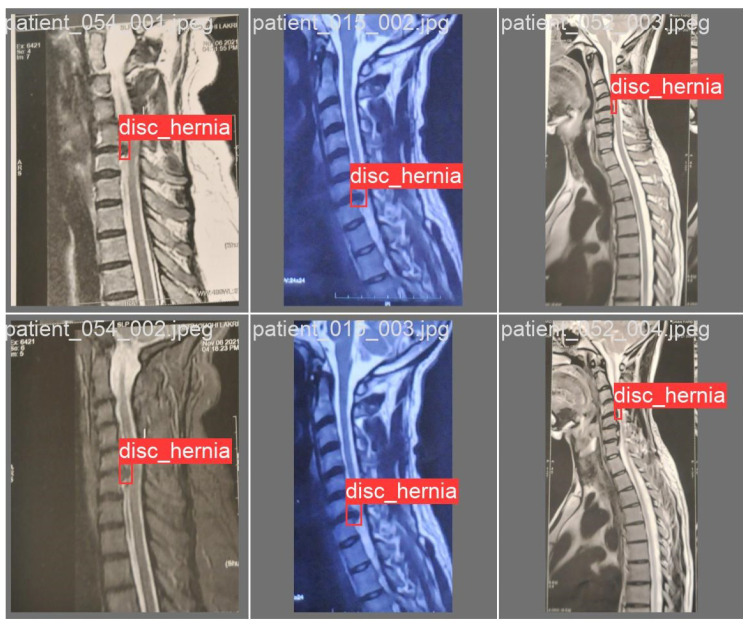
Examples from the used dataset.

The evaluation of the AI model was conducted using a held-out test set from the curated hospital dataset and a supplementary external test set to assess generalization (Figure 8). Performance was benchmarked using standard metrics, including recall, precision, F1-score, and mean Average Precision (mAP@0.5).

**Figure 9:**
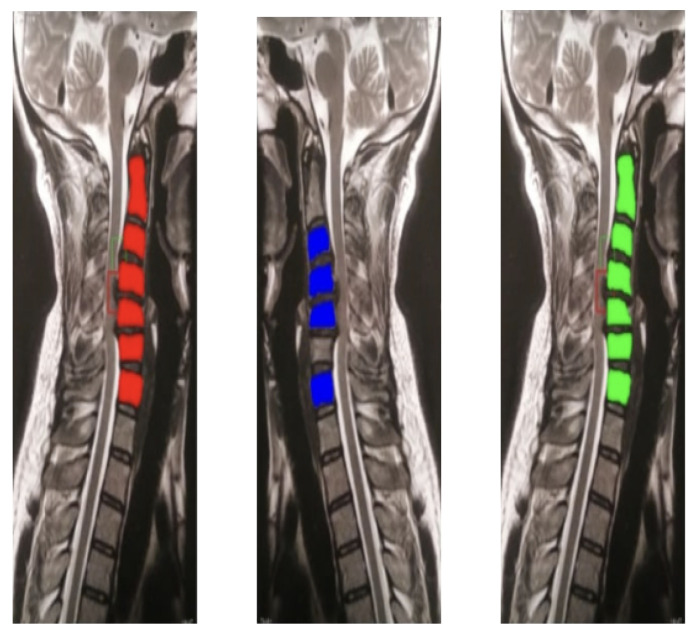
Segmentation results illustrating the mirrored image processing technique.

To further enhance the model’s robustness and accuracy, a unique mirrored image processing technique was implemented (see Figure 9). This technique addresses potential inconsistencies in the MRI data by accounting for image variations, resulting in more reliable diagnoses.

The segmentation masks are shown as follows:

- Original: segmentation mask from the standard input.
- Mirrored: segmentation mask obtained from the horizontally flipped input.
- Combined: pixel-wise ensemble of the Original and Mirrored segmentation masks, producing a unified output that improves boundary precision.

**Figure 10:**
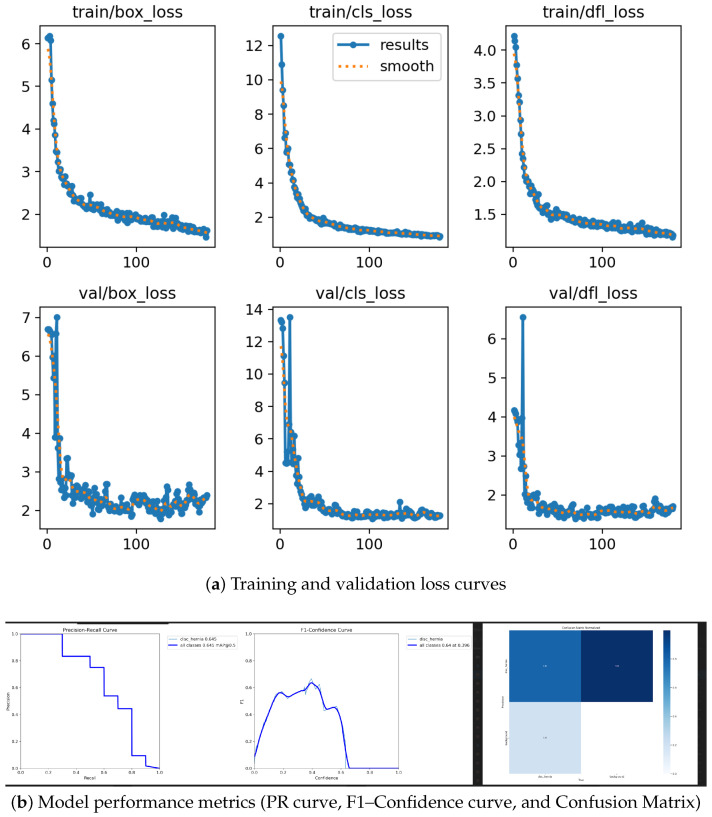
AI model evaluation metrics. (**a**) Progression of various training and validation losses over 300 epochs, indicating no significant overfitting. (**b**) Performance metrics including the Precision–Recall curve, F1—Confidence curve, and Normalized Confusion Matrix, demonstrating high diagnostic reliability.

The model’s training progression is illustrated in Figure 10, which shows that the validation metrics closely tracked the training metrics, indicating no significant overfitting.

#### 4.5.1. Disc Herniation Detection

The model’s ability to accurately identify cervical disc herniations was evaluated on an internal test cohort of 30 patients (120 slices total). To ensure a rigorous assessment of both sensitivity and specificity, the cohort was composed of 20 patients with confirmed disc herniations (80 slices, approximately 67% of the test set) and 10 patients without herniations (40 slices, approximately 33% of the test set), yielding a 2:1 herniation-to-control ratio. Table 2 summarizes the cohort composition and the mean performance metrics across the full test set.

- Performance on cases with confirmed herniations: The model was tested on a dedicated set of MRI scans from the University Hospital of Batna dataset, all of which had confirmed cervical disc herniations. In this set of known positive cases, the model successfully detected the presence of a disc herniation in 80% of the images (recall or sensitivity). For the instances where a herniation was detected by the model within this positive set, the precision reached 98% (which happened because the test data size was small, but it shows very promising results on bigger data sizes). The F1 score combining precision and recall for herniation detection on this test set was 88%.
- *Performance on cases without herniations*: To assess the model’s specificity, a supplementary test was conducted using distinct MRI scans known to have no cervical disc herniations. In this test, the model correctly identified 97% of these cases as having no herniation (specificity/accuracy on negative cases).

#### 4.5.2. Cervical Vertebrae Segmentation

**Figure 11:**
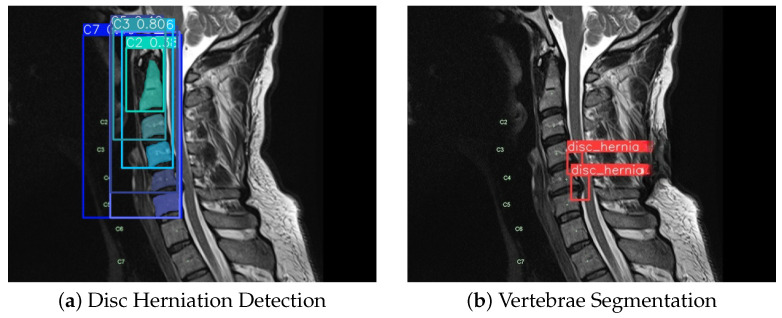
Qualitative examples of the YOLOv8 model’s performance on test images, showing (**a**) accurate detection of a disc herniation and (**b**) precise segmentation of cervical vertebrae.

The model’s precision in delineating individual cervical vertebrae (C2–C7) was measured using the mean Average Precision (mAP) at an Intersection over Union (IoU) threshold of 0.5 (mAP@0.5). This evaluation was performed on the test set of images containing confirmed herniations. The model achieved an overall mAP@0.5 of 0.995 across all targeted vertebrae (C2–C7) within these scans, see Figure 11.

#### 4.5.3. Evaluation of Mirrored Image Processing

The mirrored image processing technique serves a dual purpose within this framework. First, during model training, horizontal mirroring functions as a data augmentation strategy: by including mirror-flipped versions of training images, the model is exposed to a greater variety of anatomical orientations, improving its ability to generalize across different scan configurations and reducing sensitivity to laterality biases. Second, as a usability and robustness feature at inference time, the technique ensures that the system can correctly process MRI scans regardless of their horizontal orientation. In real-world clinical scenarios, a user may inadvertently upload an inverted or horizontally flipped MRI image; the mirroring pipeline enables the AI model to recognize and segment cervical structures reliably under these conditions. In terms of quantitative metrics, direct significant improvements on the primary reported metrics were not isolated in this evaluation. However, qualitative analysis (as depicted in Figure 9) indicated more consistent segmentation boundaries in images with slight rotational or orientational variations. The pixel-wise ensemble of the original and mirrored segmentation outputs (the “Combined” result shown in Figure 9) increased boundary delineation precision by reducing artifacts arising from asymmetric scan orientations.

## 5. Discussion

The findings from our system-level simulations and AI model evaluations suggest that this approach has the potential to advance beyond conventional cervical support methods and offers distinct advantages compared to other technology-driven neck braces.

### 5.1. Key Findings

The system-level simulations indicated the feasibility of the proposed adaptive control mechanism. The ability of the simulated system to accurately achieve commanded inflation pressures and dynamically adjust these pressures in response to mock postural changes (e.g., sustained flexion, transition to resting posture) provides foundational support for the concept of real-time adaptive cervical care. For example, the simulated corrective pressure increase in response to sustained flexion suggests a potential for the collar to actively guide users towards more neutral neck postures. These simulation outcomes, while not a substitute for physical testing, validate the core control logic and the potential for the collar to respond to dynamic user needs.

The internal review of the mobile application yielded positive initial feedback on core usability and the intuitiveness of features like the 3D visualization. This suggests the user interface design is on a viable path, although comprehensive patient-based usability testing remains crucial.

The AI model’s performance in analyzing MRI scans is a cornerstone of the proposed system. The achieved recall for detecting herniations in known positive cases (Section 4.5.1) indicates a promising capability, though further improvements to reduce the percentage of false negative rate will be essential for clinical reliability.

The model’s ability to correctly identify images without herniations, as demonstrated in preliminary tests (Section 4.5.1), offers an initial positive indication of its specificity, although this requires confirmation with a larger negative cohort.

Furthermore, the exceptional mAP@0.5 of 0.995 mAP for vertebral segmentation (Section 4.5.2) underscores the model’s proficiency in accurately delineating crucial anatomical landmarks. This high level of segmentation accuracy is vital for the system’s ability to precisely locate herniations relative to vertebral structures, which in turn informs the targeted nature of the support.

### 5.2. Comparison with Existing Approaches

The proposed system offers distinct advantages over existing cervical support solutions.

Conventional neck collars often provide generalized, static support, failing to address the specific vertebral levels implicated in conditions like cervical disc herniation, which can lead to suboptimal efficacy or discomfort. Our system, by contrast, leverages AI analysis of patient-specific MRI scans to inform personalized inflation strategies, aiming to deliver targeted therapy.

When compared with advanced AI-driven neck braces, many existing systems focus on movement assistance, general posture correction based on external sensors, or emergency protection [9,20]. These applications, while valuable, do not typically incorporate deep analysis of underlying spinal pathology from medical imaging to tailor therapeutic support to the same extent as proposed here. Our work distinguishes itself by making the MRI-derived understanding of the disc herniation central to the collar’s adaptive operation.

Furthermore, traditional technology-driven braces employing motors for active support or parallel manipulator mechanisms for precise motion control can be effective but are often characterized by bulkiness and mechanical complexity. Sensor-based braces that primarily monitor movement and gather data offer limited direct therapeutic intervention. Custom-fitted static orthoses, though initially personalized, lack the capacity to adapt to changes in a patient’s condition or activity levels over time.

The novelty of our proposed system lies in the synergistic integration of (i) AI-driven interpretation of medical images (MRI) for precise pathology localization, (ii) an adaptable (soft) inflatable collar designed for modulated support via its four-chamber system, (iii) real-time (simulated) responsiveness to user posture, and (iv) a user-centric mobile interface that facilitates control and engagement. This combination, particularly the direct link between MRI analysis and the dynamic adjustment of an inflatable support structure, represents a versatile and potentially more responsive solution for managing cervical spine issues. Our choice of a soft collar base aligns with existing research highlighting the benefits of such collars for patient comfort and compliance in managing acute cervical spine conditions [31].

### 5.3. Significance and Potential Implications

The proposed inflatable neck brace has the potential to significantly enhance the noninvasive management of cervical disc herniation. By personalizing support based on objective medical imaging and adapting to user activity, the system could offer more effective pain alleviation, promote better patient compliance through improved comfort and targeted action, and possibly contribute to accelerated recovery by reducing strain on affected areas.

The mobile application, by providing transparency into the AI’s recommendations and allowing user adjustments, empowers patients and encourages active participation in their therapy. The inclusion of a 3D preview of collar adjustments further aids patient understanding and engagement. If successfully translated into clinical practice, this technology could serve as a valuable, data-driven tool for personalized cervical care.

### 5.4. Limitations and Ethical Considerations

Despite the promising design and initial simulated results, this study has several limitations.

Importantly, this work should be framed as a proof-of-concept study. While the simulations provide a strong theoretical foundation and validate the control logic, they cannot replace the empirical data obtained from physical prototypes and human trials. Several critical gaps exist between the simulated environment and real-world application that must be addressed before clinical deployment: material fatigue and long-term durability of inflatable components under extended use, sensor noise and accuracy degradation in dynamic real-world environments with patient movement, and the lack of clinical trials necessary to establish therapeutic efficacy and definitive safety parameters.

- AI Model generalization and dataset bias: The AI model’s performance requires validation on larger, more diverse datasets covering a wider range of patient demographics, MRI scanner variations, and image qualities. The current dataset, while curated with neurosurgeon input, may contain inherent biases. Mitigating potential biases is crucial for ensuring equitable and reliable AI decision-making.
- Simulation vs. real-world performance: The system-level performance was primarily evaluated via simulation. Real-world complexities, including the nuanced biomechanics of the human neck, individual tissue compliance, subjective comfort, sensor noise in a dynamic environment, and the physical performance of actuators, may differ from the idealized simulated conditions. Simulations cannot fully replicate long-term wearability or the ease of use in daily activities.
  A fully integrated, wearable physical prototype has not yet undergone extensive testing. Therefore, crucial aspects such as material durability, actual battery life, real-world sensor accuracy, and overall user acceptance remain to be empirically evaluated.
- Clinical validation gap: The current work lacks clinical trials. The therapeutic efficacy, patient-reported outcomes, and definitive safety parameters (including optimal pressure ranges for various conditions) of the device in patients with cervical disc herniation must be established through rigorous clinical investigation.
- Ethical considerations and data security: While Firebase was employed for its security features to protect patient data, the processing of sensitive medical information inherently raises ethical concerns regarding data privacy, algorithmic transparency, and the responsibility for AI-driven therapeutic decisions. Continuous attention to robust security measures and ethical guidelines is imperative.
- System suitability: The proposed inflatable collar, while aiming for comfort, may not be suitable for all neck conditions, particularly those requiring rigid immobilization or invasive treatments.

### 5.5. Future Work

Addressing the identified limitations and advancing this technology will be the focus of future research.

- AI model: Continue to expand and diversify the MRI dataset, including a substantial number of no herniation cases, to improve model robustness and generalization.
- Physical prototype: Fabricate and rigorously test multiple iterations of a fully functional wearable prototype, focusing on sensor integration, actuator performance, material comfort and durability, and battery efficiency.
- Clinical trials: Design and conduct ethically approved clinical trials with target patient populations to systematically evaluate the device’s safety, therapeutic efficacy (pain reduction, functional improvement, etc.), and user acceptance compared to standard treatments. This will be essential for determining clinically validated pressure ranges and adaptive protocols.
- Refinement of adaptive algorithms: Leverage data from physical prototype testing and clinical trials to further refine the AI-driven adaptive algorithms for more nuanced and effective real-time support adjustments.
- Comprehensive usability and user experience: Conduct formal usability studies with patients to gather detailed feedback on the collar and mobile application, leading to iterative improvements in user experience.
- *Advanced features*: Investigate the integration of additional sensing modalities (e.g., EMG for muscle activity monitoring) or more sophisticated multi-zone inflation strategies if deemed beneficial from clinical feedback [32,33].

## 6. Conclusions

This paper presented the design, implementation, and simulation-based evaluation of an AI-guided inflatable neck brace, a novel system developed to offer personalized and adaptive cervical support [34] for individuals with disc herniations. The core of the system is an AI model trained to analyze patient MRI scans, integrated with (simulated) real-time sensor data to dynamically adjust collar inflation via a user-friendly mobile application.

Key findings from this work highlight the system’s strong potential. The developed AI model accurately detects cervical disc herniations and segments cervical vertebrae, providing a reliable anatomical foundation for personalized support. Furthermore, system-level simulations indicated the collar’s capability to accurately implement inflation commands and to responsively adapt its inflation levels based on mock postural inputs. These results underscore the feasibility of integrating AI-driven MRI analysis with dynamic collar control for tailored cervical therapy.

Future work, as detailed in Section 5.5, will focus on addressing the system’s limitations.

## Figures and Tables

**Table 1:** Key parameters for the 3D simulation environment.

Parameter	Value/Setting
Starting Time	0 units
Ending Time	100 units
Environment	Blender (for modeling), Three.js (for interaction)
Lighting	Point Light Source, 100 W
Patient Data Source	MRI Scan Analysis
Camera View	Perspective, 335.5 mm focal length

**Table 2:** Internal test cohort composition and mean performance metrics for disc herniation detection.

Group	Patients (Slices)	Proportion
Confirmed Herniation	20 (80 slices)	≈67%
No Herniation (Control)	10 (40 slices)	≈33%
Total	30 (120 slices)	100%
Mean Performance Metrics (Full Test Set)		
Precision	98%	
Recall (Sensitivity)	80%	
F1-Score	88%	

## Data Availability

The data analyzed in this study are available from the corresponding authors upon reasonable request.
